# Effects of capecitabine treatment on the uptake of thymidine analogs using exploratory PET imaging agents: ^18^F-FAU, ^18^F-FMAU, and ^18^F-FLT

**DOI:** 10.1186/s40644-016-0092-2

**Published:** 2016-10-17

**Authors:** Christopher I. McHugh, Jawana M. Lawhorn-Crews, Dipenkumar Modi, Kirk A. Douglas, Steven K. Jones, Thomas J. Mangner, Jerry M. Collins, Anthony F. Shields

**Affiliations:** 1 Cancer Biology Graduate Program, Wayne State University, Detroit, MI 48201 USA; 2 Karmanos Cancer Institute and Oncology, Wayne State University, 4100 John R., HW04HO, Detroit, MI 48201 USA; 3 Radiology, Wayne State University, Detroit, MI 48201 USA; 4 National Cancer Institute, Bethesda, MD 20892 USA

**Keywords:** PET, Oncology, Capecitabine, FLT, FMAU, FAU

## Abstract

**Background:**

A principal goal for the use of positron emission tomography (PET) in oncology is for real-time evaluation of tumor response to chemotherapy. Given that many contemporary anti-neoplastic agents function by impairing cellular proliferation, it is of interest to develop imaging modalities to monitor these pathways. Here we examined the effect of capecitabine on the uptake of thymidine analogs used with PET: 3’-deoxy-3’-[^18^F]fluorothymidine (^18^F-FLT), 1-(2’-deoxy-2’-[^18^F]fluoro-β-D-arabinofuranosyl) thymidine (^18^F-FMAU), and 1-(2’-deoxy-2’-[^18^F]fluoro-β**-**D-arabinofuranosyl) uracil (^18^F-FAU) in patients with advanced cancer.

**Methods:**

Fifteen patients were imaged, five with each imaging agent. Patients had been previously diagnosed with breast, colorectal, gastric, and esophageal cancers and had not received therapy for at least 4 weeks prior to the first scan, and had not been treated with any prior fluoropyrimidines. Subjects were imaged within a week before the start of capecitabine and on the second day of treatment, after the third dose of capecitabine. Tracer uptake was quantified by mean standard uptake value (SUV_mean_) and using kinetic analysis.

**Results:**

Patients imaged with ^18^F-FLT showed variable changes in retention and two patients exhibited an increase in SUV_mean_ of 172.3 and 89.9 %, while the other patients had changes ranging from +19.4 to -25.4 %. The average change in ^18^F-FMAU retention was 0.2 % (range -24.4 to 23.1) and ^18^F-FAU was -10.2 % (range -40.3 to 19.2). Observed changes correlated strongly with SUV_max_ but not kinetic measurements.

**Conclusions:**

This pilot study demonstrates that patients treated with capecitabine can produce a marked increase in ^18^F-FLT retention in some patients, which will require further study to determine if this flare is predictive of therapeutic response. ^18^F-FAU and ^18^F-FMAU showed little change, on average, after treatment.

**Electronic supplementary material:**

The online version of this article (doi:10.1186/s40644-016-0092-2) contains supplementary material, which is available to authorized users.

## Background

Capecitabine is a carbamate prodrug form of 5-fluorouracil (5-FU), approved for the treatment of metastatic colorectal and breast cancers, and can be used as monotherapy or in combination with other cytotoxic and targeted agents [1, 2]. Conversion to 5-FU is accomplished via the action of three enzymes: carboxylesterase, cytidine deaminase, and thymidine phosphorylase, the latter of which is found at higher concentrations in tumor cells than in normal tissue [3, 4]. Following conversion to 5-FU, anti-tumor activity is achieved via inhibition of thymidylate synthase (TS) and incorporation of 5-FU into RNA and DNA [4, 5]. Despite its widespread use, additional research is needed to explore its mechanisms of cytotoxicity, activation, metabolism, and to develop methods to monitor efficacy.

Due to its effects on thymidine synthesis and incorporation pathways, capecitabine may alter the uptake and retention of thymidine analogs used with positron emission tomography (PET) imaging and this could provide a method for assessing response and understanding drug pharmacodynamics. In part, this is due to increased expression of thymidine kinase 1 (TK1) in the salvage pathway, which is involved in the uptake and utilization of thymidine from the plasma through phosphorylation. Increased TK1 expression in tumors has been imaged with ^11^C-thymidine and thymidine analogs such as 3’-deoxy-3’-[^18^F]fluorothymidine (^18^F-FLT) [6–8]. ^18^F-FLT has been used to monitor cell proliferation [9, 10], since after uptake by tumor nucleoside transporters, ^18^F-FLT is phosphorylated by TK1, causing it to be trapped intracellularly [11, 12]. Because ^18^F-FLT is unable to incorporate into the DNA structure due to the lack of a 3’ hydroxyl, its retention principally reflects intracellular TK1 activity [13–15]. Uptake of FLT is reproducible and has been shown to be correlated with the proliferative marker Ki-67 [6, 10, 16].

1-(2’-deoxy-2’-fluoro-β-D-arabinofuranosyl) thymidine (FMAU) is another thymidine analogue that was originally introduced as an anti-viral and anti-neoplastic compound, but was later abandoned due to severe toxicity [17, 18]. More recently, FMAU has been adapted to molecular imaging [6, 19]. A key difference between FMAU and FLT is that FMAU has an intact 3’ hydroxyl group and can therefore incorporate into the DNA [20]. Furthermore, FMAU is a more potent substrate for thymidine kinase 2 (TK2), located in the mitochondria, than TK1 [18]. Unlike TK1, TK2 is constitutively expressed, with low activity in both dividing and quiescent cells [21, 22]. Accumulation of ^18^F-FMAU is higher in tumors than most healthy tissues and preclinical studies have shown that its uptake is enhanced in response to conditions that produce an increase in mitochondrial mass such as oxidative, reductive, and energy stress [23, 24]. In addition, low physiologic uptake of ^18^F-FMAU by normal bone marrow may allow it to be useful in the detection and monitoring of bone marrow metastases [19]. Further, the rapid clearance of ^18^F-FMAU from the blood in humans (90 % cleared within 10 min), allows for improved imaging in the pelvis compared to ^18^F-FLT and shortened imaging time [19, 25].

1-(2’-deoxy-2’-fluoro-β**-**D-arabinofuranosyl) uracil (FAU) is a nucleoside analog that functions as a prodrug form of FMAU [20]. Following cellular uptake of FAU, it is phosphorylated to FAU monophosphate (FAU-MP) and then converted to FMAU monophosphate (FMAU-MP) via the action of TK1 and TS, respectively [26]. FMAU-MP is then incorporated into DNA, resulting in cell death [27]. Dependence on TS for activation was designed to target FAU against malignancies with high expression of this enzyme and to avoid the neurotoxicity that resulted in the discontinuation of clinical FMAU use [17, 28–30]. High expression of TS is a major mechanism of resistance to chemotherapeutic agents such as 5-FU and capecitabine and has been associated with poor clinical outcome in breast and colorectal cancer [31–33]. Furthermore, the structure of FAU allows for its tissue distribution to be monitored using PET, and potentially serve as a technique for imaging the *de novo* TdR synthesis pathway [34, 35]. To that end, studies of ^18^F-FAU in humans and dogs found have found higher uptake in tumors than normal tissue [28, 29]. More recently, a pharmacokinetic modeling study demonstrated that the conversion of FAU to FMAU is greatly increased in tumors compared to normal tissues [36]. Although its clinical use was discontinued due to hepatoxicity, FAU may have some utility as an imaging agent.

The purpose of this study was to monitor the retention of radiolabeled fluoropyrimidines: ^18^F-FLT, ^18^F-FMAU, and ^18^F-FAU in patients with breast and gastrointestinal cancers who received capecitabine. Given the differences in metabolism for each of the tracers, the effects of capecitabine were expected to vary. The primary objective was to monitor changes in tracer uptake as measured by mean standardized uptake value (SUV_mean_) along with kinetic parameters. These parameters may provide an approximation of the physiological effect of capecitabine on tumors.

## Methods

### Radiochemistry and patient imaging

PET tracers were synthesized as previously published and patients were injected intravenously with ^18^F-FLT (range, 347–389 MBq; mean 372 MBq), ^18^F-FAU (range, 211–396 MBq; mean 346 MBq), or ^18^F-FMAU (range, 191–388 MBq; mean 339 MBq) over 60s as described [25, 37, 38]. Subjects underwent dynamic PET with a series of timed images (4×20s, 4×40s, 4×60s, and 4×180s). In patients injected with ^18^F-FLT and ^18^F-FAU, but not ^18^F-FMAU, an additional series of images was collected (8x300s). PET was conducted with a 15-cm field of view over the area of the tumors (neck, thorax, or abdomen) followed by a whole body image using an Exact/HR tomograph (Siemens Medical Solutions, Malvern, Pennsylvania, USA).

Fifteen patients with solid tumors were imaged, five with each of the fluorine-18 labeled PET tracers. Patient accrual alternated between the three agents based primarily on tracer availability. Malignancies included were breast, colorectal, gastric, and esophageal cancers (Table 1; Additional file 1: Table S1). Patients had not received therapy for at least 4 weeks prior to the first PET scan, and had not been previously treated with 5-FU, capecitabine or other fluoropyrimidines. Six of the 15 patients studied received capecitabine alone. Other patients were placed on standard regimens, which utilized radiotherapy and oxaliplatin as well as targeted agents such as lapatinib, bevacizumab, and trastuzumab (Table 1). When capecitabine was combined with other treatments they were started after the third dose of capecitabine and after completion of the final PET scan. Patients underwent imaging within one week before therapy, and again one day after the start of therapy, after receiving three doses of capecitabine. The mean time between scans was 3.7 days (range 2–7 days).

**Table 1:** Clinical patient characteristics

Patient no.	Age	Sex	Tumor type	Other therapy with initial capecitabine	Imaging tracer
1	47	F	Breast	Lapatinib	^18^F-FLT
2	65	F	Breast	None	
3	62	F	Esophageal	Radiation	
4	62	F	Colorectal	Bevacizumab, Oxaliplatin	
5	56	F	Colorectal	Oxaliplatin	
6	63	F	Breast	None	^18^F-FMAU
7	52	F	Breast	Lapatinib	
8	46	F	Breast	Lapatinib	
9	73	F	Breast	None	
10	63	F	Breast	None	
11	64	F	Breast	None	^18^F-FAU
12	62	F	Colorectal	Oxaliplatin, Bevacizumab	
13	53	F	Gastric	None	
14	49	M	Colorectal	Radiation	
15	37	M	Esophageal	Oxaliplatin, Trastuzumab	

Patient images were analyzed with PMOD (Zurich, Switzerland) software and regions of interest (ROIs) were defined in a semi-automated fashion as published [19]. ROIs were chosen in the three adjacent planes with the highest activity, using isocontours halfway between the minimum and maximum thresholds of the tumor. Tracer uptake was measured by standardized uptake value (SUV). Mean SUVs (SUV_mean_) were calculated on whole ROIs, and maximum SUVs (SUV_max_) were measured as the pixels with the most activity in the same ROIs.

### Kinetic analysis

Kinetic modeling was conducted using PMOD (Zurich, Switzerland) software as has been published previously [39]. In short, ^18^F-FLT and ^18^F-FAU time-activity curves were fitted using a 3-compartment model, which produced rate constants K1, k2, and k3. K1 (mL/g/min) represents the unidirectional transport of tracer from blood into tissue, k2 (min^−1^) represents the reverse transport, and k3 (min^−1^) characterizes phosphorylation and intracellular trapping via thymidine kinase-1 activity. The flux values for ^18^F-FLT and ^18^F-FAU were then calculated as K1 x k3/(k2 + k3). Tumor uptake values and blood tissue kinetics were interpreted with respect to the blood activity level, obtained from measurements of tracer activity within great vessels.

For ^18^F-FMAU kinetic analysis, we utilized tumor retention ratio (TRR), which has been shown to correlate strongly with compartmental-K. TRR was obtained by dividing the tumor ^18^F-FMAU activity—obtained in an image from 5 to 11 min post-injection—area under the curve (AUC) by of ^18^F-FMAU blood activity AUC. AUC values were calculated using GraphPad Prism version 6 (GraphPad Software, La Jolla, California, USA), which measures AUC using the trapezoid method. To reduce image noise, the first 5 min were omitted. Furthermore, we have previously shown that in ^18^F-FMAU blood activity decreases sharply in the first 11 min after injection, and that images taken within the 5–11 window are comparable to images from 50–60 min [19].

### Statistical considerations

The relationship of one PET parameter to another was measured using linear regression models, and the goodness of fit of these models was assessed using the r^2^ value. Regression models were fit and assessed using GraphPad Prism version 6 (GraphPad Software, La Jolla, California, USA).

## Results

### ^18^F-FLT PET imaging

**Fig. 1:**
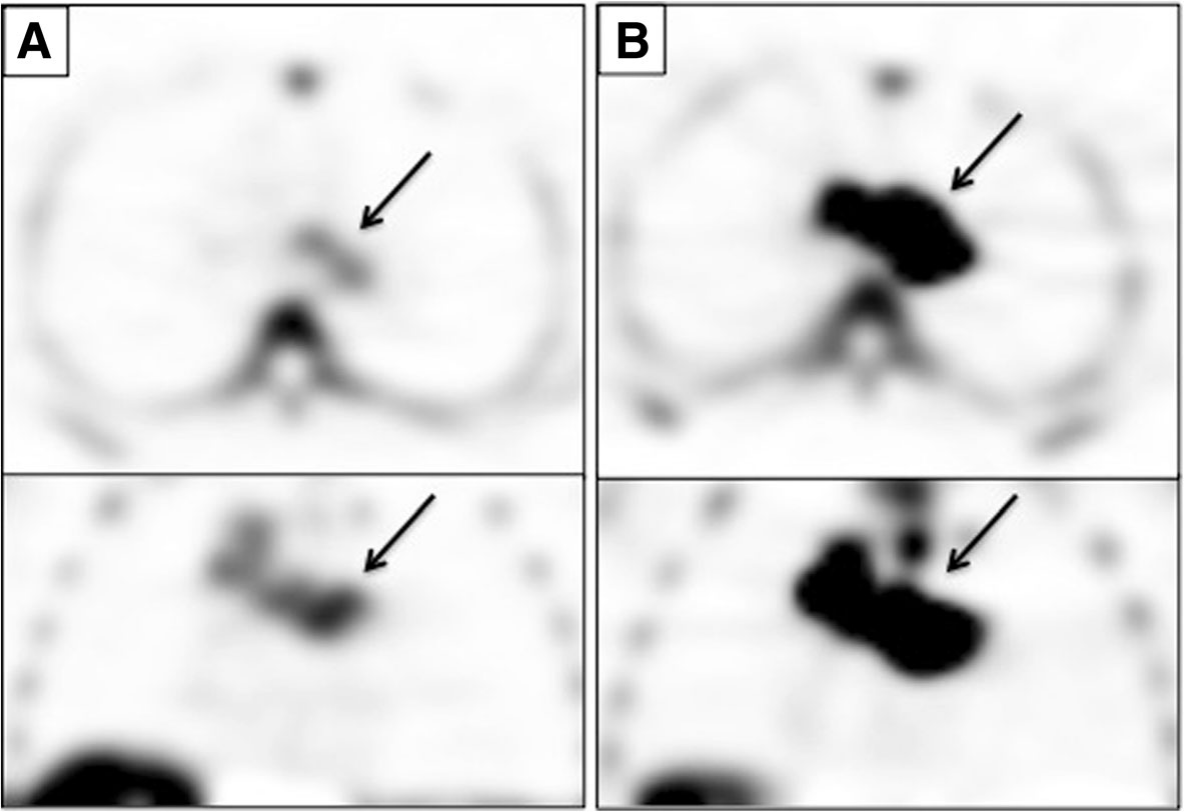
Tumor ^18^F-FLT Uptake in Patient 3. Axial (top) and coronal (bottom) ^18^F-FLT Images of a mediastinal metastasis (arrow) in a patient with esophageal cancer at baseline (**a**) and after 1 day of capecitabine therapy (**b**). Tumor SUV_mean_ increased from 4.70 to 12.80

Five patients (median age: 62) with breast, esophageal, and colorectal carcinomas were imaged with ^18^F-FLT at baseline, and then following capecitabine therapy. In addition to capecitabine, 4/5 patients underwent other anti-neoplastic therapy including: oxaliplatin, irinotecan, bevacizumab, lapatinib, and radiation after the second scan (Table 1). Variable changes in tumor activity were observed post-treatment (Table 2; Additional file 2: Table S2). Patient 3 exhibited the largest change in SUV_mean_, with an increase of 172.3 % from baseline (Fig. 1). Patient 4 also had a marked change in tracer retention, with an increase in SUV_mean_ of 89.9 % after capecitabine. The other three patients imaged had more modest changes in tumor SUV_mean_, ranging from an increase of 19.4 % to a decline of 25.4 %. Although the primary endpoint was tracer uptake as measured by SUV_mean_, the changes observed correlated with changes in SUV_max_ (*r* ^2^ = 0.98, *P* = 0.0014). Although differences in tracer flux, calculated from compartmental-K, trended with changes in tumor SUV (Table 2), changes in flux and SUV_mean_ were not correlated (*r* ^2^ = 0.57, *P* = 0.1404).

**Table 2:** Tumor retention in patients imaged with ^18^F-FLT

Patient No.	Tumor SUV_mean_			Tracer flux into tumor (cc/min)		
	Baseline	Post-treatment	% Change	Baseline	Post-treatment	% Change
1	1.97	1.58	−19.8	0.0271	0.0211	−22.1
2	1.96	2.34	19.4	0.0314	0.0526	67.5
3	4.70	12.80	172.3	0.0217	0.0796	266.8
4	2.27	4.31	89.9	0.0187	0.1090	482.9
5	1.34	1.00	−25.4	0.0267	0.0213	−20.2

### ^18^F-FMAU PET imaging

**Fig. 2:**
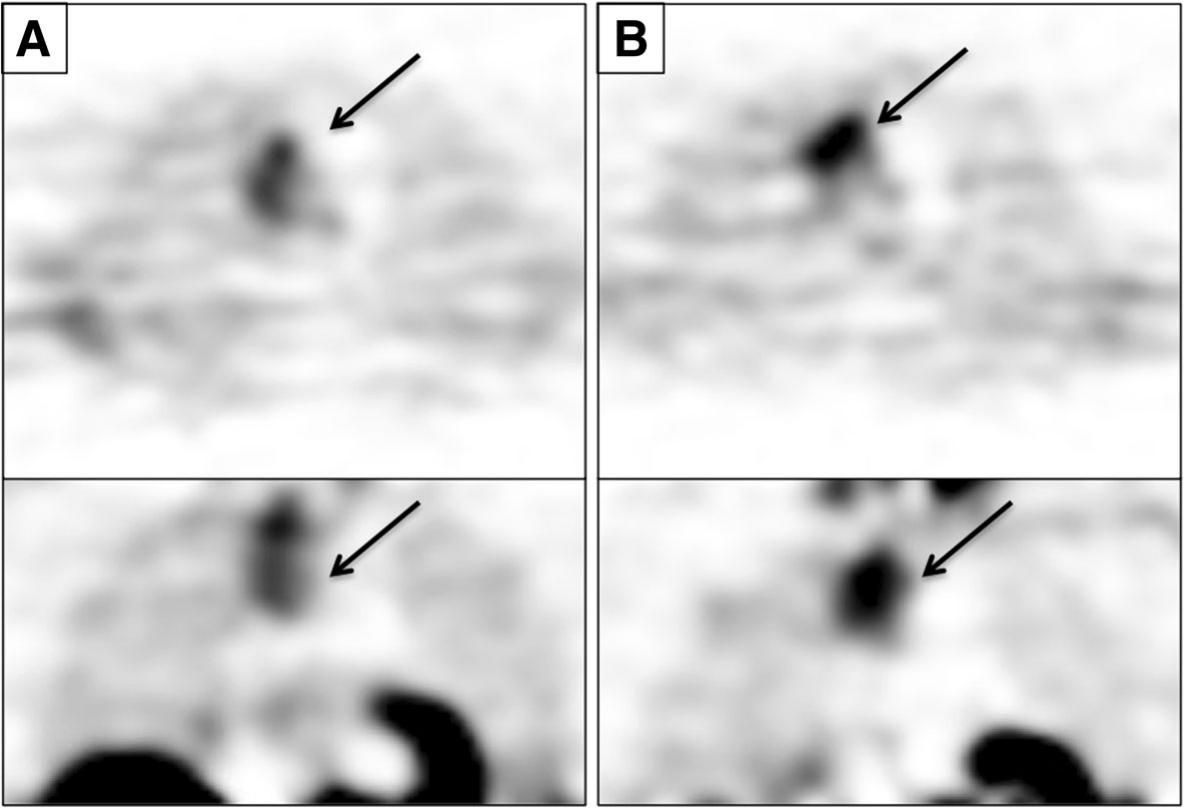
Tumor ^18^F-FMAU Uptake in Patient 7. Axial (top) and coronal (bottom) ^18^F-FMAU Images of a lung metastasis (arrow) in a patient with breast cancer at baseline (**a**) and after 1 day of capecitabine therapy (**b**). Tumor SUV_mean_ increased from 3.76 to 4.63

Five patients with breast cancer (median age: 63) were imaged with ^18^F-FMAU at baseline and following capecitabine treatment. Two patients received laptinib after the start of capecitabine (Table 1). Although tumor activity was consistently high in patients imaged with ^18^F-FMAU (median SUV_mean_ at baseline: 2.58), there was non-specific tracer uptake throughout the lungs, which gave images a ‘grainy’ appearance (Fig. 2). Following capecitabine treatment, SUV_mean_ values ranged from an increase in 23.1 % to a decline of 24.4 % from baseline, with an average change of 0.2 % (Table 3; Additional file 2: Table S2). SUV_mean_ correlated strongly with SUV_max_ measurements (*r* ^2^ = 0.95, *P* = 0.005). As mentioned, TRR was used for kinetic analysis in lieu of compartmental-K in patients imaged with ^18^F-FMAU because the rapid clearance of FMAU prevents the establishment of equilibrium between tissue compartments [19]. Similarly to what was observed in patients imaged with ^18^F-FLT, differences in SUV_mean_ and TRR after treatment trended in the same direction, but were not strongly correlated (*r* ^2^ = 0.65, *P* = 0.098).

**Table 3:** Tumor uptake in patients imaged with ^18^F-FMAU

Patient no.	Tumor SUVmean			Tumor retention ratio		
	Baseline	Post-treatment	% Change	Baseline	Post-treatment	% Change
6	4.64	5.06	9.1	3.01	3.47	15.3
7	3.76	4.63	23.1	3.56	3.9	9.6
8	1.97	2.11	7.1	2.18	2.74	25.7
9	2.58	1.95	−24.4	2.03	1.65	−18.9
10	2.14	1.84	−14.0	1.22	0.96	−21.3

### ^18^F-FAU PET imaging

**Fig. 3:**
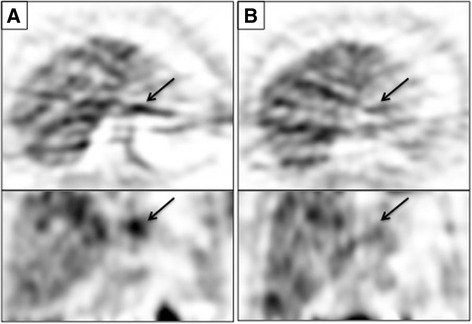
Tumor ^18^F-FAU Uptake in Patient 15. Axial (top) and coronal (bottom) ^18^F-FAU Images of a tumor of the gastroesophageal junction (arrow) at baseline (**a**) and after 1 day of capecitabine therapy (**b**). Tumor SUV_mean_ decreased from 3.47 to 2.07

Five patients (median age: 53) with breast, gastric, colorectal, and esophageal junction tumors underwent ^18^F-FAU PET scans at baseline and after capecitabine treatment. Two patients received capecitabine alone, and the remaining three also received treatment with either an antibody or radiation (Table 1). The majority of the patients showed little change in tracer uptake post-treatment (average change -10.2 %) (Table 4, Additional file 2: Table S2). Only patient 15 displayed a notable change in ^18^F-FAU retention, with a decline of 40.3 % after capecitabine (Fig. 3). Like the previous tracers, ^18^F-FAU retention was high in the kidneys and liver, but greater non-specific tissue uptake was observed compared to patients imaged with ^18^F-FLT and ^18^F-FMAU. In addition, of the tracers studied, ^18^F-FAU had the lowest tumor activity. As with ^18^F-FLT, changes in SUV_mean_ measurements correlated strongly with changes in SUV_max_ (*r* ^2^ = 0.98, *P* = 0.001). Tracer flux was calculated for 4/5 patients, with patient 11 being unevaluable due to lack of dynamic imaging. As with the previous two tracers studied herein, in patients imaged with ^18^F-FAU, tracer flux and SUV_mean_ were not significantly correlated (*r* ^2^ = 0.72, *P* = 0.1534). Furthermore, mean pretreatment ^18^F-FAU flux values were far lower than what was observed with ^18^F-FLT (0.0059 cc/min versus 0.0251 cc/min), further underscoring the low tumor accumulation of ^18^F-FAU in this patient cohort.

## Discussion

Although several radiolabeled molecules have been developed for use with PET, 2’-deoxy-2’-[^18^F]fluoro-D-glucose (^18^F-FDG) remains the principal approved compound for the detection and staging of cancer. Although ^18^F-FDG uptake correlates with general tumor metabolism, this may not accurately describe the proliferative capacity of cancers, which is a major consideration for treatment and prognosis. Further, because many chemotherapeutics used today function by impairing cellular proliferation, it is desirable to develop imaging modalities to monitor these pathways. Accordingly, we sought to examine the effect of capecitabine, a frequently used anti-neoplastic compound, on the uptake and retention of three nucleoside analogs. The goal of this study was to gain an increased understanding of the effect of capecitabine on tumor thymidine metabolism, and to assess the usefulness of these tracers in the setting of cancer treatment.

When evaluating changes in PET tracer accumulation, it is important to understand the reproducibility of such measurements in order to distinguish changes in tumor biology from simple scan-to-scan variance. A previous study in 9 non-small cell lung cancer patients found the error of ^18^F-FLT-PET to be approximately 20 % [16]. More recently, a multi-center trial examining the repeatability of PET with ^18^F-FDG in untreated patients found tumor SUV to vary between a decrease of 30 % to an increase of 40 % [40]. Although, there have been no studies examining the repeatability of imaging with ^18^F-FMAU or ^18^F-FAU, tumor retention of these tracers is lower than ^18^F-FDG, and thus, one would not expect improved reproducibility. In our study we do not think that the changes seen between PET scans reflect tumor progression, since the time between baseline and post-treatment scans ranged from 2 to 7 days. Furthermore, we do not think that a response to treatment could cause any clinical decline in the tumor, since the second scan was done a day after the start of therapy with capecitabine.

Patients imaged with ^18^F-FLT had a variable change in uptake after treatment, with two patients displaying a substantial increase in tumor retention (89.9 and 172.3 %). Since ^18^F-FLT uptake reflects cellular TK1, the large increase in SUV_mean_ indicates an upregulation of TK1 activity following capecitabine. This may be caused by the inhibitory effect of 5-FU on TS [41]. As thymidine levels drop due to TS inhibition, there is an increase in TK1 activity as cells attempt to replenish thymidine exogenously. This increase leads to a window of 1–24 h in which ^18^F-FLT uptake is significantly increased, and has been termed the ‘flare’ phenomenon [42, 43]. This effect has been observed in response to nucleoside analogs: 5-FU and gemcitabine, as well as antifolates: methotrexate and pemetrexed in preclinical models of glioma, esophageal, colon, and breast cancer [41–46].

A recent study examining the flare phenomenon in colorectal cancer patients treated with 5-FU and oxaliplatin found that ^18^F-FLT accumulation increased in all patients 24 h after treatment, but increases in tumor SUV > 45.8 % were associated with poor treatment outcomes [47]. It is possible that a large flare may suggest that cancers are able to successfully compensate for drug-induced TS inhibition. Therefore, a large increase in ^18^F-FLT retention may be a negative indicator of therapy response. Conversely, the absence of change in ^18^F-FLT retention in the remaining three patients may suggest that patient tumors were unable to effectively adapt to capecitabine treatment. Alternatively, the absence of a flare could be due to upregulation of intracellular TS levels leading to drug resistance, or inefficient conversion of capecitabine to 5-FU [48].

Subjects imaged with ^18^F-FMAU demonstrated little change in tracer retention after treatment. The average change in tumor SUV_mean_ was 0.18 % (range -24.4 to 23.1) (Table 3). Previous studies have shown increases in ^18^F-FMAU retention in response to oxidative, reductive, and energy stresses due to upregulation of mitochondrial TK2 levels [49]. Furthermore, it has been shown that anti-cancer agents can lead to an increase in in mitochondrial mass during apoptosis [50, 51]. Interestingly, patients imaged with ^18^F-FMAU had the highest baseline tumor uptake: 2.58 versus 2.45 in patients scanned with ^18^F-FLT and 1.99 patients scanned with ^18^F-FAU. These findings suggest that while tumor cells are under a high basal level of cellular stress, this is not increased significantly by short-term capecitabine treatment.

Similar to patients imaged with ^18^F-FMAU, patients scanned with ^18^F-FAU demonstrated little change in tracer retention after capecitabine (Table 4), with an average change in SUV_mean_ of -10.2 %. No difference in measurement may be due to several factors, including elevated tumor TS. As discussed, high tumor TS is a common mechanism of treatment resistance in breast and colorectal cancers [33]. In this case TS will continue to convert FAU-P to FMAU-P, with treatment having a negligible effect on this process. One patient demonstrated a decrease of 40.3 % in tumor SUV_mean_ from baseline in response to capecitabine. This may be evidence of inhibition of TS by capecitabine, given that TS required for retention of ^18^F-FAU [26]. It is worth noting, however, that tumor activity was lowest in patients imaged with ^18^F-FAU, suggesting a low level of tumor specificity for this tracer.

**Table 4:** Tumor retention in patients imaged with ^18^F-FAU

Patient no.	Tumor SUVmean			Tracer flux into tumor (cc/min)		
	Baseline	Post-treatment	% Change	Baseline	Post-Treatment	% Change
11	1.03	1.06	2.9	No Dynamic Images		
12	1.05	0.87	−17.1	0.0032	0.0019	−40.6
13	2.57	2.15	−16.3	0.0058	0.0055	−5.2
14	1.82	2.17	19.2	0.0108	0.0158	46.3
15	3.47	2.07	−40.3	0.0039	0.0029	−25.6

Major limitations of this study included small sample sizes and heterogenous patient cohorts. Patients enrolled in this study had several different malignancies and received varied treatment regimens (Table 1 and Additional file 1: Table S1). The duration of capecitabine treatment after the second PET scan was inconsistent between individuals and the majority of subjects (9 of 15) were administered other anti-neoplastic therapy in addition to capecitabine. For these reasons, we are unable to correlate our imaging findings to patient response to capecitabine and therefore our results should be considered observational.

## Conclusions

In this exploratory study, we sought to monitor the response of patient tumors to capecitabine, a commonly used chemotherapeutic, using three experimental imaging tracers: ^18^F-FLT, ^18^F-FMAU, and ^18^F-FAU. Patients who underwent PET with ^18^F-FAU and ^18^F-FMAU showed little change, on average, after treatment. However, in-line with similar studies, we observed that patients treated with capecitabine can produce a marked increase in ^18^F-FLT retention in some patients. Further studies are warranted to determine if this effect could be used as an early biomarker for therapeutic efficacy.

## Additional files

Additional file 1: Table S1. Additional Patient Information. (XLSX 10 kb)
Additional file 2: Table S2. Patient Image Data. (XLSX 19 kb)

## Abbreviations

5-FU: 5-Fluorouracil
AUC: Area under the curve
FAU: 1-(2’-deoxy-2’-fluoro-β**-**D-arabinofuranosyl) Uracil
FAU-P: FAU Monophosphate
FDG: 2’-deoxy-2’-fluoro-D-glucose
FLT: 3’-deoxy-3’-fluorothymidine
FMAU: 1-(2’-deoxy-2’-fluoro-β-D-arabinofuranosyl) Thymidine
FMAU-P: FMAU Monophosphate
PET: Positron emission tomography
ROI: Region of interest
SUV: Standardized uptake value
TK1: Thymidine kinase 1
TRR: Tumor retention ratio
TS: Thymidylate synthase
